# People with diagnosed HIV infection not attending for specialist clinical care: UK national review

**DOI:** 10.1186/s12879-015-1036-3

**Published:** 2015-08-06

**Authors:** Hilary Curtis, Z. Yin, K. Clay, A. E. Brown, V. C. Delpech, E. Ong, on behalf of the BHIVA Audit and Standards Sub-Committee

**Affiliations:** British HIV Association, c/o Mediscript, 1 Mountview Court, 310 Friern Barnet Lane, London, N20 0LD UK; Public Health England, London, UK; Heartlands Hospital, Birmingham, UK; Department of Infection & Tropical Medicine, Royal Victoria Infirmary, Newcastle, UK

## Abstract

**Background:**

Regular clinical care is important for the well-being of people with HIV. We sought to  audit and describe the characteristics of adults with diagnosed HIV infection not reported to be attending for clinical care in the UK.

**Methods:**

Public Health England (PHE) provided clinics with lists of patients diagnosed or seen for specialist HIV care in 2010 but not linked to a clinic report or known to have died in 2011. Clinics reviewed case-notes of these individuals and completed questionnaires. A nested case–control analysis was conducted to compare those who had remained in the UK in 2011 while not attending care with individuals who received specialist HIV care in both 2010 and 2011.

**Results:**

Among 74,418 adults living with diagnosed HIV infection in the UK in 2010, 3510 (4.7 %) were not reported as seen for clinical care or died in 2011. Case note reviews and outcomes were available for 2255 (64 %) of these: 456 (20.2 %) remained in the UK and did not attend care; 590 (26.2 %) left UK; 508 (22.6 %) received care in the UK: 73 (3.2 %) died and 628 (27.8 %) had no documented outcome. Individuals remaining in the UK and not attending care were more likely to be treatment naïve than those in care, but duration since HIV diagnosis was not significant. HIV/AIDS related hospitalisations were observed among non-attenders.

**Conclusion:**

Retention in UK specialist HIV care is excellent. Our audit indicates that the ‘true’ loss to follow up rate in 2011 was <2.5 % with no evidence of health tourism. Novel interventions to ensure high levels of clinic engagement should be explored to minimise disease progression among non-attenders.

## Background

The life expectancy of patients living with HIV has substantially improved since the advent of antiretroviral therapy (ART) in the mid-1990s. If people are diagnosed promptly, linked into HIV care, receive ART when indicated, maintain good adherence on therapy, and receive care regularly in a specialist clinic their life expectancy appears to be comparable to that of the general population [1, 2]. Furthermore, virological suppression on ART offers significant potential public health benefits in terms of prevention of onward transmission of HIV [3]. UK national guidelines recommend routine screening of patients at risk of HIV acquisition [4–6] and regular care for all HIV positive individuals [7] to facilitate optimal clinical outcomes, including for example 2–4 clinic visits annually in ART-naïve patients.

HIV treatment and care is provided free of charge to UK residents through specialist HIV outpatient clinics within the National Health Service, funded through taxation. Previous studies have shown that about 5 % of individuals aged 15 years and above who receive HIV care in a given year have no linkable care or death report for the following year, and that cumulatively, an estimated one in five HIV positive patients may be lost to follow-up over a 5 year period [8, 9]. However, the extent to which this reflects out-migration as compared with non-attendance for care among individuals remaining in the UK is unclear. We asked specialist HIV clinical services to review records of individuals known to be HIV positive in 2010 who had no linkable care or death report during 2011, to estimate the numbers who had remained living in the UK without attending for HIV care and who had left the UK, and to compare the non-attending group with individuals who continued receiving care.

## Methods

Individuals attending specialist HIV care in England, Wales and Northern Ireland (E, W, NI) are reported annually for public health monitoring purposes via the Survey of Prevalent HIV Infections Diagnosed (SOPHID) to Public Health England (PHE, formerly Health Protection Agency), using soundex code (a 4-character coding of surname), sex, date of birth and postcode of residence to provide a unique identifier. This preserves confidentiality while enabling linking of different reports over time or across services relating to the same individual. New HIV diagnoses are similarly reported to PHE with the same variables to identify individuals. Using methods previously described [8] PHE surveillance reports of adults (16 or over) seen for specialist HIV care or newly diagnosed with HIV in 2010 were linked to corresponding SOPHID and Health Protection Scotland (HPS) reports of individuals attending specialist HIV care in 2011 anywhere in the UK. Death notifications are reported directly to PHE by clinics and are supplemented by death notifications from the Office of National Statistics; these notifications were also linked to surveillance reports of adults seen for specialist care or newly diagnosed with HIV in 2010.

Patients were categorised by whether they were diagnosed before, or during 2010 (“newly diagnosed”). Records from adults who were seen for care in 2010 but had no care or death reports in 2011 were sent securely to their care providers. Records from adults who were newly diagnosed in 2010 but had no care or death reports in both 2010 and 2011 were assigned to their clinic of diagnosis if this was a SOPHID-reporting service providing specialist HIV care, or otherwise to the nearest SOPHID-reporting service.

During October-December 2012, the British HIV Association (BHIVA) requested specialist HIV services to review case records of these individuals and complete online questionnaires. Anonymous summary data were sought for all individuals, comprising age, gender, ethnicity, country of birth, mode of HIV acquisition, whether registered with a general practitioner (GP), whether there was a record of the person having attended clinic at least once during each of the years 2010, 2011 and 2012 up to the date of submission, and patient location (in or outside UK) in 2011. To allow for fuller analysis while not unduly burdening respondents, extended data were requested for a randomly selected sample of five or 10 cases per clinical service depending on its HIV caseload (fewer or greater than 500 patients). This dataset included: year of HIV diagnosis; ART history (naïve or having been prescribed ART whether or not discontinued prior to when the person was last seen); CD4 T-cell count when last measured; clinician’s assessment of attendance history and ART adherence if ART-experienced; whether the individual had declined ART during the year up to when s/he was last seen; whether attempts were made to contact the individual and/or his/her GP to encourage re-attendance; and clinical status in 2012 for those who re-attended that year. It also included information not presented here: year of arrival in the UK if born abroad; setting of HIV diagnosis (whether in UK and if so type of clinic); lowest recorded CD4 T-cell count; attendance and ART adherence; methods of contact if this was attempted; and social, psychological and economic issues.

HPS surveillance reports were not linked for individuals seen or diagnosed during 2010 in Scotland, but BHIVA asked Scottish HIV clinical services to complete the same questionnaires for individuals they had seen for care in 2010 whom they believed had not been seen for HIV care in their own or any other UK clinic during 2011.

A nested case–control analysis was conducted to determine if patients’ characteristics (age, sex, ethnicity and likely mode of HIV acquisition) are associated with not receiving care (outcome). Cases were individuals who had attended for HIV specialist care in 2010 and had been reported via the online questionnaire as remaining in the UK, but not attending specialist care during 2011. Controls were individuals who received specialist HIV care in both 2010 and 2011. Cases and controls were matched by PHE centre/country in E, W, NI with an 1:10 case–control ratio.

To investigate the impacts of clinical factors (ART status and CD4 cell count) on retention, an extended case–control analysis was conducted among cases and controls with information on HIV diagnosis date, ART history and CD4 cell count in 2010 available. Thus, potential risk factors for being lost to follow up were patients’ characteristics and these three clinical factors (ART history, period of HIV diagnosis and CD4 cell count).

STATA 12.0 (Stata Corp., College Station, Texas, USA) was used for analyses. Univariate and multivariate logistic regression models were used to identify significant risk factors in the case–control analyses. Proportions were presented where adults with missing information were included in analyses. Testing values and all confidence intervals (CI) are at the 95 % significant level.

Ethical approval and informed consent was not required as this study was a clinical audit [10] based on data routinely collected at HIV clinics. Furthermore, clinic data collected for public health purposes are securely kept in strict confidence by PHE and subject to regulations made under Section 251 of the National Health Service Act 2006. No names were collected for this study.

## Results

**Fig. 1:**
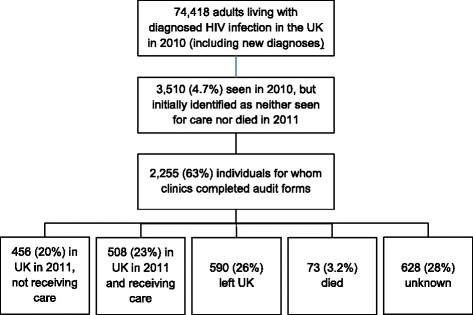
Flowchart showing study inclusion and outcomes for 2255 adults (16 or over) with diagnosed HIV infection

PHE and HPS received surveillance reports of 74,418 adults attending for HIV specialist care and/or newly diagnosed with HIV infection in the UK in 2010. PHE provided clinics in E, W, NI with details of 3,452 individuals with no linked 2011 care or death report, and clinics completed audit questionnaires for 2,197 (63.6 %) of these. Comparison of the distributions of age, sex, ethnicity and route of HIV infection of the 3,452 individuals with the 2,197 for whom questionnaires were completed suggested minimal response bias, with no statistically significant differences (data not shown). Clinics in Scotland completed questionnaires for a further 58 patients seen in 2010 and believed not to have received care in 2011. This resulted in a total of 3,510 individuals in the UK who were seen for care or newly diagnosed in 2010 and initially identified as not seen for care in 2011, with audit questionnaires completed for 2,255 of these (see Fig. 1). Characteristics of these individuals were as shown in Table 1.

**Table 1:** Characteristics of all individuals initially identified as not seen for care in 2011 for whom audit forms were completed, and of those reported by the clinic as remaining in the UK and not receiving care

Characteristic	Number (%) of individuals for whom audit questionnaire completed	Number (%) of individuals remaining in UK and not receiving care during 2011	Number (%) of individuals remaining in UK and not receiving care during 2011 with complete demographic information
Total	2255 (100)	456 (100)	308 (100)
Sex:			
Male	1,290 (57.2)	175 (38.4)	178 (57.8)
Female	838 (37.2)	272 (59.6)	130 (42.2)
Trans-gender	5 (0.2)	1 (0.2)	0
Not known/stated	122 (5.4)	8 (1.8)	0
Age:			
16–19	9 (0.4)	2 (0.4)	1 (0.3)
20–29	310 (13.7)	76 (16.7)	45 (14.6)
30–39	765 (33.9)	167 (36.6)	115 (37.3)
40–49	717 (31.8)	144 (31.6)	97 (31.5)
50–59	274 (12.2)	53 (11.6)	41 (13.3)
60+	103 (4.6)	10 (2.2)	9 (2.9)
Not known/stated	77 (3.4)	4 (0.9)	0 (0)
Ethnicity:			
Black-African	1,028 (45.6)	214 (46.9)	172 (55.8)
White	846 (37.5)	179 (39.3)	136 (44.2)
Other	284 (12.6)	58 (12.7)	0 (0)
Not known/stated	97 (4.3)	5 (1.1)	0 (0)
Birthplace:			
UK	600 (26.6)	176 (38.6)	114 (37.0)
Outside UK	1,383 (61.3)	246 (53.9)	176 (57.1)
Not known/stated	272 (12.1)	34 (7.5)	18 (5.8)
^a^Likely mode of HIV acquisition:			
Heterosexual sex	1,320 (58.5)	277 (60.7)	202 (65.6)
Sex between men	726 (32.2)	148 (32.5)	106 (34.4)
Injecting drug use	64 (2.8)	14 (3.1)	0 (0)
Receipt of blood/blood products	22 (1.0)	1 (0.2)	0 (0)
Vertical infection	16 (0.7)	6 (1.3)	0 (0)
Other	11 (0.5)	0 (0.0)	0 (0)
None stated	134 (5.9)	16 (3.5)	0 (0)

^a^More than one mode of HIV acquisition could be selected

Clinic-reported outcomes are shown in Fig. 1. Just under half of individuals who were initially identified as not seen for care in 2011 may have remained living in the UK while not receiving care. This group comprised 456 (20.2 %) individuals recognised as having been in the UK and out of care (see Table 1) and a further 628 (27.8 %) with unknown outcomes (of which: not known whether in UK 578, 25.6 %; not identifiable from information provided by PHE 50, 2.2 %). The remainder were accounted for by departure from the UK (590, 26.2 %), having received care (508, 22.6 %) or having died (73, 3.2 % - this is additional to individuals for whom PHE had identified a linked death report). Strong evidence for 427 individuals having left the UK included documented plans to leave or a request for a clinician letter to an overseas care provider (385 cases), information from immigration authorities (28) or prisons (2), and the patient contacting the clinic from abroad (7) or reporting having been abroad in 2011 when returning to care in 2012 (5). In 71 cases the evidence was less clear, eg simply a record that the person had left the UK, and for 92 individuals this question was not answered.

Extended data were provided for 230 individuals who remained in the UK and who were not in receipt of care in 2011. Of these, 112 (48.7 %) had a history of irregular attendance and there were significant adherence concerns for 27 (23.9 % of 113 with ART experience), but only 30 (13.0 %) had declined ART in the year up to when they were last seen. Of the 230 individuals, 97 (42.2 %) re-presented for care during 2012 prior to data collection in October-December, including 28 (12.2 %) with symptomatic illness among whom there were 9 inpatient admissions and 7 AIDS-defining diagnoses. Clinics reported attempts to contact 183 (79.6 %) of these 230 individuals to encourage them to return to care, but although 124 (53.9 %) were registered with a GP who was aware of their HIV status, clinics contacted the GP in only 69 (30.0 %) cases.

Of 456 individuals who were recognised as having remained in the UK but not attended for specialist care in 2011, 308 had complete demographic information and were included in regression analyses. Of these 308 individuals, extended data were available for 136 individuals. In univariate analysis, individuals who received specialist HIV care in 2010 and remained in the UK but not in receipt of such care in 2011 were more likely to be female, heterosexually exposed (cf male homosexual), black-African (cf white), younger, recently diagnosed, ART-naïve and with a low CD4 T-cell count than individuals who received specialist HIV care in both 2010 and 2011 (Table 2). In multivariable analysis based on demographic summary data, black-African ethnicity and younger age remained significantly associated with not being in receipt of care in 2011. When the multivariable analysis was expanded to include factors in the extended dataset (year of HIV diagnosis, ART status and CD4 T-cell count) being ART-naïve and low CD4 T-cell count were the only factors significantly associated with not being in receipt of care in 2011, while age and ethnicity ceased to be significant.

**Table 2:** Associations from univariate and multivariable logistic regression models between demographic and clinical factors and retention in care in 2011

	Univariate analysis:	Multivariable analysis of summary data for 308 cases, 3080 controls:	Multivariable analysis of extended data for 136 cases, 2313 controls:
	Odds ratio (95 % CI)	Odds ratio (95 % CI)	Odds ratio (95 % CI)
Sex:			
Male	Ref	Ref	Ref
Female	**1.30 (1.02–1.64)**	0.83 (0.61–1.14)	1.19 (0.73–1.93)
Age:			
16–19	0.92 (0.11–7.63)	0.79 (0.09–6.66)	1.34 (0.12–14.51)
20–29	Ref	Ref	Ref
30–29	0.76 (0.52–1.09)	0.70 (0.48–1.01)	0.89 (0.49–1.62)
40–49	**0.52 (0.36–0.76)**	**0.48 (0.33–0.71)**	0.89 (0.48–1.62)
50–59	**0.57 (0.37–0.90)**	**0.56 (0.35–0.88)**	0.54 (0.24–1.21)
60+	**0.36 (0.17–0.75)**	**0.39 (0.18–0.82)**	0.73 (0.23–2.32)
Ethnicity:			
White	Ref	Ref	Ref
Black-African	**1.67 (1.32–2.11)**	**1.66 (1.14–2.43)**	1.66 (0.92–2.97)
Likely mode of HIV acquisition:			
Male homosexual	Ref	Ref	Ref
Heterosexual	**1.52 (1.19–1.94)**	**1.14 (0.75–1.73)**	1.33 (0.69–2.58)
Year of diagnosis:		Not in summary data	
2008 or earlier	Ref		Ref
2009–10	**2.23 (1.52–3.25)**		1.03 (0.65–1.62)
ART before 2012:		Not in summary data	
History of ART	Ref		Ref
No ART	**9.15 (6.47–12.93)**		**12.12 (7.87–18.64)**
Last CD4 T-cell count in 2011 2010 (cells/mm^3^)		Not in summary data	
0–200	Ref		Ref
201–350	0.79 (0.41–1.51)		0.70 (0.34–1.45)
351–500	**0.39 (0.20–0.74)**		**0.24 (0.11–0.52)**
500+	**0.36 (0.20–0.66)**		**0.26 (0.13–0.52)**

## Discussion

Among adults seen for care or diagnosed with HIV in 2010 without a linkable care or death report for 2011, clinics recognised only a fifth (20.2 %) as having remained in the UK but not in receipt of specialist HIV care, while a further 27.8 % had unknown outcomes and may have been in the same situation. Of the remainder, 26.2 % had left the UK, 22.6 % were reported to have received care and 3.2 % had died. Previous studies have shown that for a given year no linkable care or death report can be found for about 5 % of adults who attended for specialist HIV care in the preceding year [8, 9]. Our study suggests that non-attendance for care among individuals remaining in the UK accounts for at most about a half of this, even if those with unknown outcomes are assumed to have been out of care in the UK. This represents an excellent rate of retention in care within the UK.

Although this finding is encouraging, non-attendance for care among individuals with diagnosed HIV remains a public health concern in terms of both disease progression and potential onward transmission of infection, especially in view of unsuppressed viraemia in the absence of ART. We were not able to investigate transmission risks, but of 230 individuals remaining in the UK and not receiving care in 2011, 28 (12.2 %) re-attended with symptomatic illness in 2012 including 9 requiring inpatient care and 7 with AIDS-defining diagnoses. While it is not possible to calculate incidence rates from our data, this appears substantially higher than in the UK-CHIC cohort of patients with established HIV infection (Sabin C, personal communication), illustrating the risk of disease progression among individuals not attending for care.

In a multivariable case–control analysis, individuals remaining in the UK who received specialist HIV care in 2010 but not in 2011 were more likely to be ART naïve and to have a low CD4 T-cell count than those who received care in both years, although the CD4 finding may simply be a reflection of non-use of ART. Duration since HIV diagnosis was not significant in this analysis, suggesting that non-attendance remains an ongoing risk among individuals not on ART. Black-African ethnicity has previously been shown to be associated with loss to follow up from UK HIV clinical care [8, 9]. We found that this is not solely due to out-migration from the UK since in multivariable analysis based on demographic factors, individuals remaining in the UK but not receiving care were more likely to be black-African.

Out-migration from the UK accounted for over a quarter of individuals lacking a linked care report for 2011 or death report. As we have reported previously [11] this mainly involved previous long term residents, and does not indicate use of specialist HIV care services by short-term visitors.

According to clinic-reported outcomes, 508 (22.6 %) individuals for whom PHE had not identified a linked 2011 care or death report had received HIV care in the UK that year, and 73 (3.2 %) had died. Some of these discrepancies may reflect mistaken assumptions, for example if a patient was believed to have transferred his/her care to a different clinic but had not in fact attended there. However, 163 individuals were reported to have attended the *same* clinical service in 2011 as in 2010; this may be explained by incomplete reporting to PHE, changes in personal identifiers and limitations of the data linking method.

In most cases HIV specialist services took active steps to re-engage with patients whom they recognised as not being in receipt of care, but these services only involved the GP in 69 of 124 cases in which the latter was aware of the patient’s HIV status. This may reflect specific historical circumstances in much of the UK, where specialist HIV care is delivered via genitourinary medicine clinics set up to provide highly confidential care for people with sexually transmitted infections. However it represents a missed opportunity to collaborate to enable more effective access to care.

Limitations of our study include reliance on case note information collected retrospectively from clinics. This led to considerable uncertainties and may possibly have resulted in misclassification, reflecting the fact that clinics often have limited information about people who do not attend for care. Outcomes were unknown for 628 (27.8 %) of cases and, as stated above, clinics may have incorrectly reported some individuals as having received care, eg in a mistaken belief that they had transferred to a different clinic. Completed audit forms were obtained for only 2,197 (63.6 %) of 3,452 individuals initially identified via record linkage as not seen for care. It is possible that clinics may have been more likely to respond in cases where they had better information, but we did not detect response bias based on demographic factors. Also, we only examined non-retention in care from 2010 to 2011 and return to care in 2012, rather than cumulative movement in and out of care over multiple years. This means the number of people living in the UK with diagnosed HIV who are not receiving care cannot be estimated from our data. A previous study found that cumulatively 19 % of individuals seen for care during 1998–2006 became lost to follow up based on SOPHID record linking [8], but did not include clinic-held information such as out-migration from the UK. In addition PHE data shows that over 80 % of individuals in England have a CD4 measurement within three months after initial HIV diagnosis [12], indicating that the vast majority become linked into a specialist HIV care service.

## Conclusions

Our study confirmed excellent retention in care among UK adults with HIV, but also the high risk of disease progression among non-attenders. Public health initiatives aimed at maintaining those at greatest risk of suboptimal attendances in care remain essential.

## Abbreviations

AIDS: Acquired immunodeficiency syndrome
ART: Antiretroviral therapy
BHIVA: British HIV Association
CD4: CD4+ T-helper lymphocyte
CI: Confidence interval
E, W, NI: England, Wales and Northern Ireland
GP: General (medical) practitioner
HIV: Human immunodeficiency virus
HPS: Health Protection Scotland
PHE: Public Health England
SOPHID: Survey of Prevalent HIV Infections Diagnosed
UK: United Kingdom
